# COVID-19 and acute juvenile paracoccidioidomycosis coinfection

**DOI:** 10.1371/journal.pntd.0008559

**Published:** 2020-08-10

**Authors:** Priscila Marques de Macedo, Dayvison Francis Saraiva Freitas, Andrea Gina Varon, Cristiane da Cruz Lamas, Livia Cristina Fonseca Ferreira, Andrea d’Avila Freitas, Marcel Treptow Ferreira, Estevão Portela Nunes, Marilda Mendonça Siqueira, Valdiléa G. Veloso, Antonio Carlos Francesconi do Valle

**Affiliations:** 1 Evandro Chagas National Institute of Infectious Diseases, Fiocruz, Rio de Janeiro, Brazil; 2 Oswaldo Cruz Institute, Fiocruz, Rio de Janeiro, Brazil; Karolinska Institutet, SWEDEN

## Case presentation

**Fig 1:**
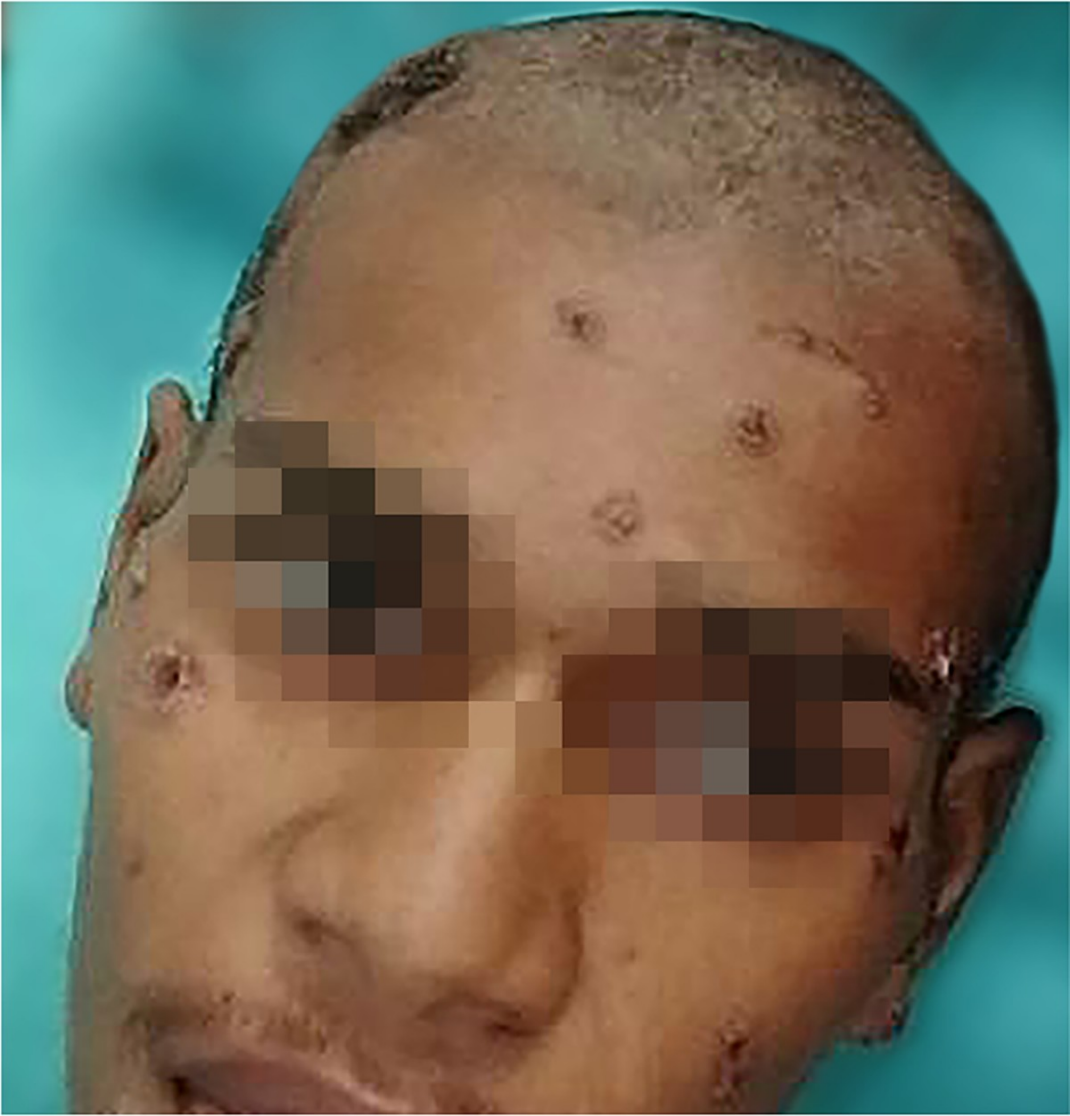
Ulcerated skin lesions covered by crusts on the patient’s face and scalp.

A 19-year-old male patient was admitted to the Evandro Chagas National Institute of Infectious Diseases, Fiocruz, on 10 March 2020 complaining of an 8-month history of progressive weight loss; multiple cervical, axillary, and inguinal lymph node enlargements; abdominal distension; and disseminated cutaneous lesions (Fig 1).

**Fig 2:**
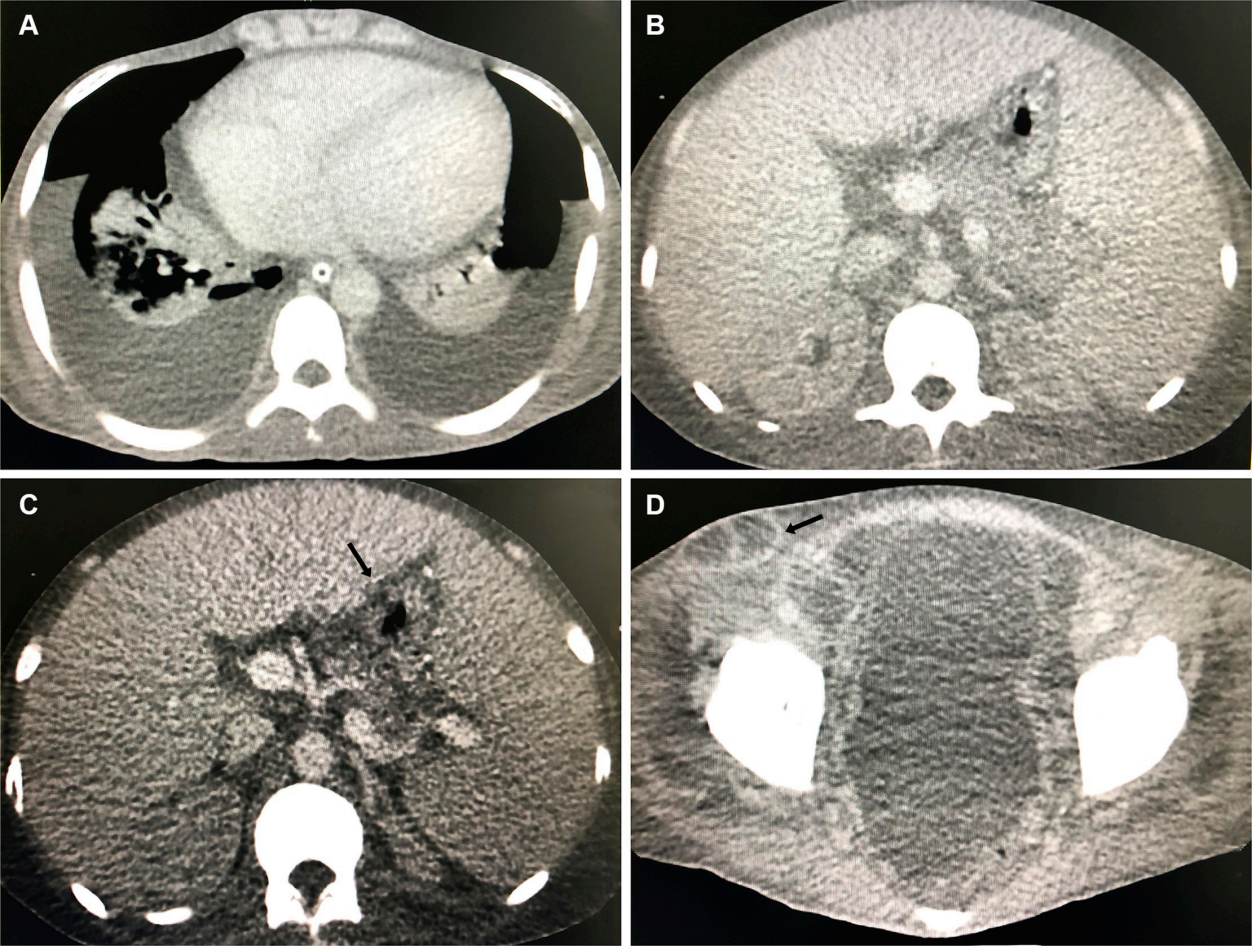
Computerized tomography images showing (A) pleural and pericardial effusion, right pulmonary consolidation; (B) hepatosplenomegaly; (C) liquefaction of peritoneal lymph nodes (black arrow); and (D) ascites and liquefaction of inguinal lymph nodes (black arrow).

A histopathological examination of the left inguinal lymph node revealed chronic granulomatous lymphadenitis with the presence of multiple budding fungal structures typical of *Paracoccidioides* spp. in silver staining. Because he presented an important left inguinal lymph node enlargement with fluctuation, its content was aspirated for relief and diagnostic confirmation. This showed typical fungal forms on microscopy, and culture was positive for *Paracoccidioides* spp. Laboratory analyses showed hemoglobin 5.8 g/dL (reference value [RV]: 13–18 g/dL); leukocytosis 13,630/mm^3^ (RV: 4,200–9,000/mm^3^) with predominance of eosinophils 17% (RV: 1%–7%); platelet count 410,000/mm^3^ (RV: 150,000–450,000/mm^3^); and normal serum biochemistry, except for low albumin levels 1.17 g/dL (RV: 3.4–5 g/dL). ELISA anti-HIV was negative, and basal cortisol levels were within normal ranges. Specific serum antibodies against *Paracoccidioides* spp. in Ouchterlony immunodiffusion test (ID) were detected (1:512). Computerized tomography (CT) images presented pleural and pericardial effusion, multiple mediastinal and peritoneal lymph node conglomerates, ascites, as well as hepatosplenomegaly (Fig 2).

The patient was hospitalized in a single room with airborne precautions until tuberculosis coinfection was ruled out. Amphotericin B lipid complex was started (5 mg/kg per day) and was well tolerated. The patient presented with some complications related to paracoccidioidomycosis (PCM), including severe protein and caloric malnutrition, intestinal subocclusion demanding nasogastric tube and total parenteral nutrition, anasarca due to hypoalbuminemia, pleural effusion worsening requiring thoracocentesis (1,100 mL of a transudative pleural fluid), and staphylococcal skin abscesses, which were drained and treated with intravenous vancomycin.

**Fig 3:**
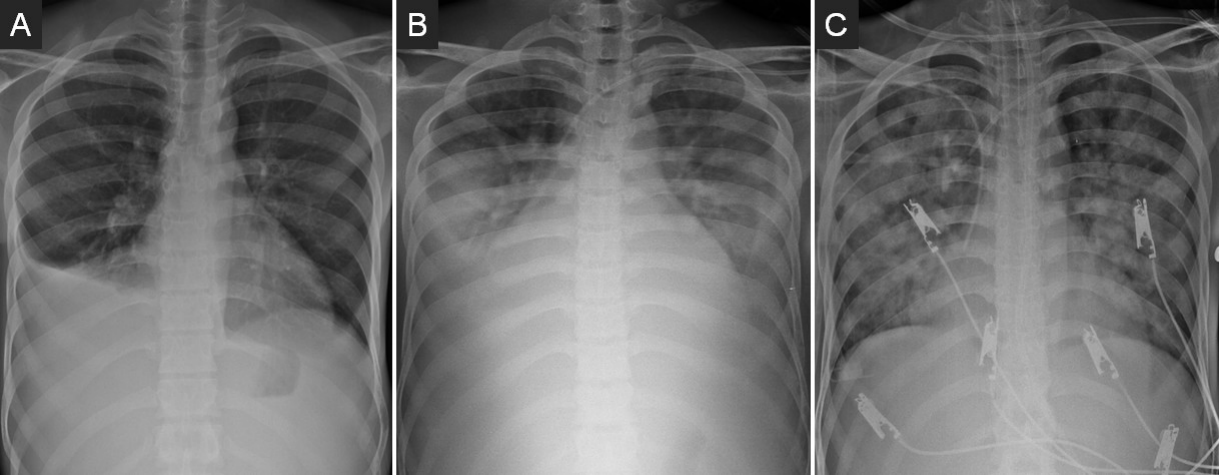
Chest radiographies presenting (A) right basal consolidation, pleural effusion (admission), (B) bilateral pleural effusion (day 21), and (C) extensive bilateral interstitial infiltrate with diffuse areas of consolidation (day 26).

On the 15th day of hospitalization, the patient presented a single episode of low fever (37.9°C) without other symptoms. Four days later (day 19), he presented two episodes of high fever (40°C), which were attributed to a catheter-related bloodstream infection. The central venous catheter was removed after serial blood cultures. The patient became afebrile. Chest radiography presented similar changes previously ascribed to his underlying PCM condition (Fig 3A and 3B). As fever reocurred 3 days later (day 22), piperacillin–tazobactam was started empirically. Blood cultures were negative, and respiratory symptoms were not present so far. On the 26th day of hospitalization, the patient presented dyspnea of sudden onset at rest and tachypnea (50 breaths per minute); oxygen saturation [SpO2] plummeted to 70%. Immediate endotracheal intubation and mechanical ventilation was provided. The patient was conducted to the intensive care unit (ICU), where he quickly evolved to shock, acute renal failure, and acute respiratory distress. Real-time reverse-transcription PCR (RT-PCR) (Biomanguinhos kit [E+P1], Fiocruz, Brazil) of nasopharyngeal secretion and bronchoalveolar fluid tested positive for severe acute respiratory syndrome–related coronavirus 2 (SARS-CoV-2). Serial CT could not be provided because of the patient’s clinical instability, but chest radiography evolved into a diffuse pattern (Fig 3C) 10 days after onset of coronavirus disease 2019 (COVID-19) symptoms. Fig 3 shows the radiological findings over time during hospitalization.

Lymphopenia and most of the available presumptive biomarkers for diagnosis of COVID-19 were not detected, except for higher levels of C-reactive protein (CRP), which was present since the patient’s admission (Table 1).

**Table 1:** Longitudinal data of the patient’s available biomarkers during hospitalization.

Biomarker	Reference Value	Admission	Day 15	Day 19	Day 22	Day 26	Day 4 ICU
WBC	4,200–9,000/mm^3^	13,630	14,210	9,370	7,340	21,560	16,540
Neutrophils	1,470–6,750/mm^3^	9,268	9,805	7,027	6,018	15,102	13,232
Eosinophils	42–630/mm^3^	2,317	1,563	749	0	862	496
Basophils	0–90/mm^3^	0	0	0	0	0	0
Lymphocytes	924–4,770/mm^3^	1,363	2,131	1,311	1,027	5,174	2,646
Monocytes	210–1,080/mm^3^	681	710	281	293	431	165
Platelets	150,000–450,000/mm^3^	410,000	321,000	249,000	315,000	371,000	259,000
CRP	0–0.3 mg/dl	9.16	9.87	10.82	9.43	19.96	31.57
LDH	85–227 U/L	119	NA	NA	NA	NA	177
Troponin	<40 ng/L	NA	NA	NA	NA	NA	NA
D-dimer	<600 ng/mL	NA	NA	NA	NA	NA	NA
Urea	15–38 mg/dl	27	62	44	25	47	132
Creatinine	0.7–1.3 mg/dl	0.85	0.85	0.58	0.57	0.81	2.72

Abbreviations: CRP, C-reactive protein; ICU, intensive care unit; LDH, lactate dehydrogenase; NA, not available; WBC, white blood cell

Epidemiological investigation revealed the existence of one potential index case. A maintenance worker who was responsible for cleaning the patient’s room the week before onset of the patient’s symptoms tested positive for SARS-CoV-2. Sequentially, three people from the nursing staff who worked shifts with the index case also tested positive. Furthermore, 2 weeks after onset of the patient’s symptoms, two attending physicians—the consultant staff and a first-year resident—also tested positive.

## Ethical statements

The Research Ethics Committee of the Evandro Chagas National Institute of Infectious Diseases (INI/Fiocruz) approved this study (CAAE 18524919.1.0000.5262). The patient’s mother signed a consent form authorizing description of the case and the release of the patient’s photographs.

## Case discussion

SARS-CoV-2 is the etiological agent of the ongoing pandemic of COVID-19, which emerged in December 2019 in Wuhan, China [1,2]. In Brazil, the first confirmed case of COVID-19—a 61-year-old man who had recently arrived from Lombardy, Italy—was registered on 26 February 2020 in São Paulo [3]. The progression of the epidemic to regions where people in social vulnerability live is a worrisome problem in countries with great social inequalities, such as Brazil.

PCM is a systemic fungal disease occurring in Latin America and more prevalent in South America [4]. Infection starts in the lungs after inhalation of *Paracoccidioides* spp. conidia present in the soil of endemic areas and can progress to disease, which manifests in two clinical forms. The most prevalent is the chronic form (adult type), accounting for 80% of PCM cases, which occurs mostly in rural workers who reactivate fungal endogenous foci, mainly in the lungs, later in life. The other form is acute (juvenile type), which occurs primarily in young patients with progressive mononuclear phagocytic system involvement, resulting in many complications, including death [5,6]. Several predisposing factors for PCM may be related to poverty. PCM fulfills WHO’s neglected tropical disease (NTD) criteria, but it has not been included in the WHO NTD list so far [7].

There is a high risk of nosocomial infection with SARS-CoV-2. Several cases of infection among healthcare professionals have been reported, as they have frequent exposure to hospitalized patients infected with SARS-CoV-2 [8,9,10]. Also, healthcare workers are exposed in the community and can transmit SARS-CoV-2 to hospitalized patients. The recommendations on the transmission-based precautions for COVID-19 in hospital settings include contact and droplet precautions, as well as airborne precaution for aerosol-generating procedures. Wong and colleagues suggest that nosocomial transmissions can be prevented through vigilant basic infection control measures, including wearing of surgical masks and hand and environmental hygiene [11]. Despite all precautions, cases can still occur, as SARS-CoV-2 is a very contagious microorganism. Vanhems and colleagues reported a fast-spreading nosocomial cluster of seven patients infected with COVID-19 and one healthcare worker in a geriatric unit, reinforcing the contagiousness of SARS-CoV-2 in healthcare settings. The existence of supershedders who could facilitate cluster emergence was also suggested [12].

PCM can represent a risk factor for severe COVID-19 cases. In the chronic PCM form, frequent tobacco abuse, chronic obstructive pulmonary disease, and pulmonary alterations caused by the infection lead to tissue fibrosis, justifying this increased risk. These patients are usually rural workers who live in the countryside and were recently affected by COVID-19 epidemics in Rio de Janeiro State. The juvenile PCM type, although rarer, has presented increasing cases in urban areas of Rio de Janeiro State [13]. This is a more severe clinical form, whose immunosuppression and complications could impose a greater risk if associated with SARS-CoV-2 infection.

Although pulmonary alterations are scarcely seen in advanced phases of acute PCM, radiological findings such as consolidations and pleural effusion can occur in some cases, as observed in the admission images of our patient. Chest radiographic findings of 64 patients with COVID-19 included consolidations (47%), followed by bilateral ground glass opacities (33%) and, rarely, pleural effusion (3%) [14]. The occurrence of some overlapping radiological findings between PCM and COVID-19 can delay the diagnosis of viral pneumonia coinfection.

In general, the clinical forms of PCM exhibit dichotomous T-helper responses: whereas patients with the chronic form still demonstrate a preserved T-cell response, as indicated by skin tests, the acute form has abundant antibodies but marked depression of cell-mediated immunity. The acute form exhibits T-helper 2 (Th2) cytokine production with high interleukin (IL)-4, IL-5 and IL-9 levels, usually present in critically ill PCM patients, regardless of the clinical form of the disease [15]. This complex immunological profile, present in severe acute PCM cases, in association with malnutrition and other PCM complications, probably contributed to the progression to severe COVID-19 presentation in this case. Notably, secondary hemophagocytic lymphohistiocytosis (sHLH), occurring in severe forms of COVID-19, and acute PCM share some similar clinical and laboratory findings that should be mentioned. Table 2 details comparative findings between these two conditions and the patient’s data.

**Table 2:** Comparative clinical and laboratory parameters between sHLH, acute PCM, and the patient’s data.

Clinical and Laboratory Aspects	HScore^1^		Acute PCM	This Patient
	Parameter	Number of Points	Parameter	Parameter
Fever (°C)	<38.4	0	Usually present	40
	38.4–39.4	33		
	>39.4	49		
Organomegaly	None	0	Hepatomegaly and/or splenomegaly often present	Hepatomegaly and splenomegaly
	Hepatomegaly or splenomegaly	23		
	Hepatomegaly and splenomegaly	38		
Number of cytopenias*	One lineage	0	Anemia often present Leukocytosis/leukopenia can occur	Anemia
	Two lineages	24		
	Three lineages	34		
Triglycerides (mmol/L)	<1.5	0	Usually normal	0.73
	1.5–4.0	44		
	>4.0	64		
Fibrinogen (g/L)	>2.5	0	Not routinely tested	Not available
	≤2.5	30		
Ferritin (ng/ml)	<2,000	0	Usually high	886
	2,000–6,000	35		
	>6,000	50		
Serum aspartate aminotransferase (IU/L)	<30	0	Usually normal High in severe liver involvement	56
	≥30	19		
Hemophagocytosis on bone marrow aspirate	No	0	Absent	Not available
	Yes	35		
Known immunosuppression†	No	0	Usually absent	Absent
	Yes	18		

^1^The HScore generates a probability for the presence of sHLH. HScores greater than 169 are 93% sensitive and 86% specific for HLH. Bone marrow hemophagocytosis is not mandatory for a diagnosis of HLH [16,17].

*Defined as either hemoglobin concentration of 9.2 g/dL or less, a white blood cell count of 5,000 cells per mm^3^ or less, platelet count of 110,000 platelets per mm^3^ or less, or all of these criteria combined [16,17].

^†^HIV positive or receiving long‐term immunosuppressive therapy [16,17].

Abbreviations: HLH, hemophagocytic lymphohistiocytosis; PCM, paracoccidioidomycosis; sHLH, secondary HLH

Because our patient presented some clinical findings shared by acute PCM and sHLH, meeting the score number for sHLH, it is not possible to rule out that this inflammatory condition has also occurred. Furthermore, the cytokine storm syndrome and immunosuppression occurring in severe COVID-19 cases combined with an already-impaired immune response may have justified the poor prognosis [16,17,18].

**Key learning points**

- We report the first case of SARS-CoV-2 nosocomial infection in a patient with acute juvenile PCM, highlighting patients with PCM as an at-risk population for severe COVID-19.
- Pulmonary findings and complications of PCM may hinder and retard the specific diagnosis and the clinical management of COVID-19.
- The case reported herein shows the harmful potential that COVID-19 can represent for vulnerable populations suffering from severe endemic mycoses.
- The authors reinforce the need for more attention to NTDs in the context of the COVID-19 pandemic.
